# Supplementary material for: Reconciling Mining with the Conservation of Cave Biodiversity: A Quantitative Baseline to Help Establish Conservation Priorities
Source: PLoS One. 2016 Dec 20;11(12):e0168348. doi: 10.1371/journal.pone.0168348 (PMC5173368; doi:10.1371/journal.pone.0168348)
Supplement: S1 Dataset — (ZIP) [file pone.0168348.s002.zip › Taxa/Serra Norte/SN_2013/N4E/Lista N4E-16.pdf]

CAVIDADE N4E-0016

Classe	Ordem	Fam/Outros	Gên/Outros	Espécie	S-U	U-S	Zon.
Annelida	Oligochaeta			jovem		2	E
Arachnida	Acari	(Oribatida)		jovem	X		
Arachnida	Acari	Trombiculidae		sp.39		X	P
Arachnida	Amblypygi	Charinidae	*Charinus*	sp.1		1	P
Arachnida	Amblypygi	Phrynidae	*Heterophrynus*	*longicornis*	X		
Arachnida	Araneae	Araneidae	*Alpaida*	aff. *negro*		X	E
Arachnida	Araneae	Barychelidae		jovem		1	E
Arachnida	Araneae	Drymusidae	*Drymusa*	*spelunca*	X	X	E/P
Arachnida	Araneae	Linyphiidae		jovem	X		
Arachnida	Araneae	Nesticidae		jovem		X	E/P
Arachnida	Araneae	Ochyroceratidae	*Ochyrocera*	sp.1	X	X	E/P
Arachnida	Araneae	Oonopidae		jovem		X	E
Arachnida	Araneae	Pholcidae	*Leptopholcus*	sp.1		X	E
Arachnida	Araneae	Pholcidae	*Metagonia*	sp.	X		
Arachnida	Araneae	Prodidomidae		sp.	X		
Arachnida	Araneae	Salticidae		sp.4	X	X	E
Arachnida	Araneae	Scytodidae	*Scytodes*	sp.	X		
Arachnida	Araneae	Theridiidae	*Theridion*	sp.2		X	E
Arachnida	Araneae	Theridiosomatidae	*Plato*	sp.1	X	X	E/P
Arachnida	Araneae	Zodariidae	*Tenedos*	sp.	X		
Arachnida	Opiliones	Stygnidae	*Protimesius*	*gracilis*		1	P
Arachnida	Palpigradi			jovem	X		
Arachnida	Pseudoscorpiones	Chthoniidae	*Pseudochthonius*	sp.1		X	E
Chilopoda	Geophilomorpha	Ballophilidae	*Ityphilus*	sp.2	X	1	E
Chilopoda	Scolopendromorpha	Scolopendridae	*Rhoda*	*thayeri*	X	1	E
Diplopoda	Polydesmida	Pyrgodesmidae		sp.1		X	P
Entognatha	Collembola	Isotomidae		sp.1		X	P
Entognatha	Collembola	Paronellidae		sp.1		X	E/P
Entognatha	Collembola	Sminthuroidea		sp.2	X	X	P
Entognatha	Diplura	Campodeidae		sp.1	X	X	E
Entognatha	Diplura	Projapygidae		sp.1	X	X	P
Insecta	Blattodea			jovem	X		
Insecta	Coleoptera	Elateridae		jovem	X		
Insecta	Diptera	Chironomidae		sp.		X	E
Insecta	Diptera	Psychodidae	Phlebotominae	sp.		X	P
Insecta	Diptera	Psychodidae	Psychodinae	sp.		X	E
Insecta	Heteroptera	Reduviidae	*Zelurus*	sp.	X	1	E
Insecta	Homoptera	Cixiidae		jovem	X	X	E
Insecta	Homoptera	Cixiidae		sp.1		X	E/P
Insecta	Homoptera	Cixiidae		sp.3		X	E
Insecta	Hymenoptera	Formicidae	*Acromyrmex*	sp.1		X	E
Insecta	Hymenoptera	Formicidae	*Camponotus*	crassus	X		
Insecta	Hymenoptera	Formicidae	*Camponotus*	sp.1		X	E/P
Insecta	Hymenoptera	Formicidae	*Gnamptogenys*	*striatula*	X		
Insecta	Hymenoptera	Formicidae	*Neivamyrmex*	sp.1		X	E
Insecta	Hymenoptera	Formicidae	*Odontomachus*	*haematodus*	X		
Insecta	Hymenoptera	Formicidae	*Odontomachus*	sp.1		X	E
Insecta	Hymenoptera	Formicidae	*Pachycondyla*	*constricta*	X		
Insecta	Hymenoptera	Formicidae	*Pachycondyla*	sp.2		X	E
Insecta	Isoptera	Termitidae	*Nasutitermes*	sp.	X	X	E
Insecta	Lepidoptera	Tineoidea		sp.	X	X	
Insecta	Orthoptera	Phalangopsidae	*Phalangopsis*	sp.	X	1	E
Insecta	Psocoptera	Epipsocidae	*Epipsocus*	sp.2	X	X	E
Insecta	Thysanura	Nicoletiidae		jovem	X	X	E
Gastropoda	Pulmonata	Systrophiidae	*Happia*	sp.	X	X	E

Symphyla		Scutigerellidae		sp.		X	E
Amphibia	Anura			sp.	1		E
Mammalia	Chiroptera			sp.	5		P
